# Strain-Dependent Recognition of a Unique Degradation Motif by ClpXP in *Streptococcus mutans*

**DOI:** 10.1128/mSphere.00287-16

**Published:** 2016-12-07

**Authors:** Biswanath Jana, Liang Tao, Indranil Biswas

**Affiliations:** Department of Microbiology, Molecular Genetics and Immunology, University of Kansas Medical Center, Kansas City, Kansas, USA; University of Michigan

**Keywords:** ClpXP, motif, *Streptococcus mutans*, adaptor proteins, regulated proteolysis

## Abstract

*Streptococcus mutans*, a dental pathogen, has a remarkable ability to cope with environmental stresses. Under stress conditions, cytoplasmic proteases play a major role in controlling the stability of regulatory proteins and preventing accumulation of damaged and misfolded proteins. ClpXP, a well-conserved cytoplasmic proteolytic system, is crucial in maintaining cellular homeostasis in bacteria. ClpX is primarily responsible for recognition of substrates and subsequent translocation of unfolded substrates into the ClpP proteolytic compartment for degradation. In *Escherichia coli*, ClpX recognizes distinct motifs present at the C-terminal end of target proteins. However, recognition sequences for ClpXP in other bacteria, including *S. mutans*, are not known. In this study, using two-dimensional (2D) polyacrylamide gel electrophoresis (PAGE) analysis, we have identified several putative substrates for *S. mutans* ClpXP. SsbA, which encodes a small DNA binding protein, is one such substrate that is degraded by ClpXP. By sequential deletions, we found that the last 3 C-terminal amino acids, LPF, are sufficient for ClpXP-mediated degradation. Addition of LPF at the C-terminal end of green fluorescent protein (GFP) rendered the protein completely degradable by ClpXP. Alterations of this tripeptide motif impeded ClpXP-mediated degradation. However, recognition of LPF by ClpXP is highly specific to some *S. mutans* strains (UA159, UA130, and N3209) since not all *S. mutans* strains recognize the motif. We speculate that an adaptor protein is involved in either substrate recognition or substrate degradation by ClpXP. Nevertheless, this is the first report of a unique recognition sequence for ClpXP in streptococci.

**IMPORTANCE** Regulated proteolysis in bacteria is an important biological process that maintains protein homeostasis. ClpXP, an intracellular proteolytic complex, is the primary protease that is responsible for protein turnover. While the substrates for ClpXP were identified in *Escherichia coli*, the substrates for vast majority of bacteria are currently unknown. In this study, we identified a unique substrate for ClpXP-mediated degradation in *Streptococcus mutans*, a dental pathogen. We also found that a small motif composed of 3 amino acids is sufficient for ClpXP-mediated degradation. Identification of this motif will clearly help us to understand the pathogenesis of this organism and other related pathogens.

## Abstract (precis)

Regulated proteolysis in bacteria is an important biological process that maintains protein homeostasis. ClpXP, an intracellular proteolytic complex, is the primary protease that is responsible for protein turnover. While the substrates for ClpXP were identified in *Escherichia coli*, the substrates for vast majority of bacteria are currently unknown. In this study, we identified a unique substrate for ClpXP-mediated degradation in *Streptococcus mutans*, a dental pathogen. We also found that a small motif composed of 3 amino acids is sufficient for ClpXP-mediated degradation. Identification of this motif will clearly help us to understand the pathogenesis of this organism and other related pathogens.

## INTRODUCTION

Proteolysis plays a major role in maintaining the cellular proteome by removing undesired proteins in response to external signals (1). From bacteria to humans, proteolysis is very important for cellular function and protein quality control. In bacteria, regulated proteolysis is an energy-dependent mechanism that is required for numerous cellular processes, including responses to various stresses and DNA damage (for recent reviews, see references 2 and 3). Regulated proteolysis requires stringent selection of the substrate and is carried out by various proteolytic complexes. ClpXP is one such complex that is composed of two proteins: ClpX, which is an AAA+ ATPase, and ClpP peptidase. ClpP alone is unable to degrade the unfolded or misfolded proteins and requires the assistance of an ATPase component such as ClpX that recognizes the substrate proteins by identifying specific short unstructured peptide sequences present at the N-terminal region or the C-terminal region (4, 5). The function of these ATPases is to unfold and translocate the substrates into the ClpP degradation chamber, where the substrate proteins are cleaved into small peptides (6–9).

The ClpXP proteolytic system has been best studied in *Escherichia coli*. In this bacterium, ClpXP recognizes peptide sequences that contain the SsrA tag at the C terminus (10). The SsrA tag is attached to the incomplete nascent polypeptide during protein synthesis by the transfer-messenger RNA (tmRNA) system and allows recognition of aberrantly made or incomplete proteins for degradation (11). The *E. coli* SsrA tag is 11 amino acids long; however, only the last three amino acids (LAA) are the key determinants for recognition by ClpX (12, 13). Often, adapter proteins further enhance specificity of proteolysis mediated by ClpXP. In *E. coli*, SspB is an adaptor protein that specifically binds to the SsrA tag by recognizing the frontal part of the tag and aids degradation by ClpXP (14). In addition to the SsrA tag, *E. coli* ClpXP can recognize other proteins that possess two alanine residues at the C terminus (15). Furthermore, *E. coli* ClpXP can recognize several other sequence tags present at either the C-terminal end or the N-terminal end. For example, a six-residue tag (RRKKAI) present at the C terminus of MuA, a phage transposase, is recognized and degraded by ClpXP (15, 16). Similarly, ClpXP can recognize at least three types of N-terminal motifs, whose lengths can vary significantly (15). The N-terminal motifs that are recognized by ClpXP have no sequence similarity to the C-terminal motifs.

*Streptococcus mutans*, a Gram-positive bacterium that is responsible for human dental plaque formation, possesses five ATPases: ClpB, ClpL, ClpC, ClpE, and ClpX (17). Among these, only three ATPases, ClpC, ClpE, and ClpX, interact with ClpP and have the distinct tripeptide motif required for interaction with ClpP to form the functional Clp proteolytic complex (18). In *S. mutans*, *clpX*-deficient or *clpP*-deficient strains generated pleiotropic effects, including a growth defect, clumping, reduced autolysis, a defect in biofilm formation, sensitivity to various stress conditions, and attenuated virulence (19–21). Furthermore, ClpXP is also involved in competence development and bacteriocin production. Surprisingly, both the *clpX* and *clpP* mutant strains displayed improved growth capability under acid stress conditions and showed enhanced viability during long-term survival (20). ClpXP has a pleiotropic effect in *S. mutans*; however, the natures of the substrates or the sequences recognized by ClpXP have not been systematically identified, except for a few target proteins such as Spx and IvrR and SsrA-tagged substrates (22–24). In fact, besides *E. coli*, the exact sequence motifs for ClpXP recognition have not been verified in other organisms.

In this study, we used a proteomic approach to identify several putative substrates for *S. mutans* ClpXP. In particular, we found that two single-stranded DNA binding proteins (SsbA and SsbB) are the bona fide substrates of ClpXP-mediated proteolysis. By generating various deletion derivatives, we narrowed down the possible residues of the recognition sequence to the last three residues. Addition of these three residues to green fluorescent protein (GFP) resulted in the GFP being targeted by ClpXP. We further found that any alteration in the tripeptide motif affects the substrate recognition, indicating that all 3 amino acids are important for the recognition. This tripeptide motif is highly specific for *S. mutans* since ClpXP from other bacteria does not recognize the motif.

## RESULTS

### ClpX-mediated protein turnover *in S. mutans*.

To understand the role of ClpX in protein turnover in the cell under nonstressed conditions, a two-dimensional gel electrophoresis (2-DGE) approach was used to compare the proteomes of a wild-type (UA159) strain to those of its isogenic *ΔclpX*-mutant (IBSJ4) strains. Crude cellular lysates from UA159 and IBSJ4 were prepared from mid-exponential-phase cultures and subjected to proteomic analysis. Approximately 560 well-resolved protein spots could be detected in the pI range of 5.0 to 8.2 by silver staining (see Fig. S1 in the supplemental material). Comparison of the proteomes from UA159 and IBSJ4 revealed that at least 53 proteins had altered levels of expression, with a fold change value of 3.0 (*P* value of ≤0.05). Among the differentially expressed proteins, 27 were upregulated and 26 were downregulated in the IBSJ4 (*ΔclpX*) strain relative to those in the UA159 strain (see Table S2 in the supplemental material). We were able to excise only 37 of 53 protein spots. We then determined the identities of those selected spots by mass fingerprinting after in-gel trypsin digestion. We could determine the identity of only 17 spots unambiguously (Table S3). Among the upregulated proteins, the spot that showed the highest fold change value (7.9×) corresponded to an autolysin (AltA) encoded by SMU.689. Two other protein spots corresponding to DivIVA (SMU.557) and SsbA (SMU.1859) were also significant since the relative abundances of these two spots were above 3-fold compared to that of the wild-type strain. Since the peptide coverage for these three proteins was relatively high, we considered these proteins to be putative targets for ClpXP. Proteins that were downregulated in the IBSJ4 strain included UvrA (SMU.1851) and LacA (SMU.1496). The relative abundance differences for these two proteins were above 3-fold. As expected, we found ClpX only in the proteome of the wild-type strain and not in the IBSJ4 proteome.

10.1128/mSphere.00287-16.4 Figure S1  Two-dimensional gel electrophoresis of total cellular proteins extracted from *S. mutans* UA159 (wild type) (A) and its isogenic *clpX* mutant (B). Cells were grown to the mid-exponential-growth phase and were harvested. Total cellular lysates were prepared in a buffer containing 10 mM Tris-Cl (pH 7.4) and 0.3% sodium dodecyl sulfate (SDS). Protein concentrations in the samples were determined using the BCA assay, and equal amounts of samples were subjected to two-dimensional gel electrophoresis, which was performed according to the carrier ampholine method of isoelectric focusing in glass tubes with 12.5 g of each sample that was spiked with 50 ng of tropomyosin (arrowhead) (33 kDa; pI, 5.2) as an internal standard. A 10% SDS slab gel electrophoresis procedure was carried out as the second dimension. The gels were stained with a special silver stain that is compatible with mass-spectrometry analysis. At least four independent 2D gels were run using samples isolated from two separate cultures, and a representative gel image is shown for each strain. Download Figure S1, PDF file, 0.1 MB. Copyright © 2016 Jana et al. 2016 Jana et al. This content is distributed under the terms of the Creative Commons Attribution 4.0 International license.

### ClpX recognizes both DivIVA and SsbA.

**FIG 1:**
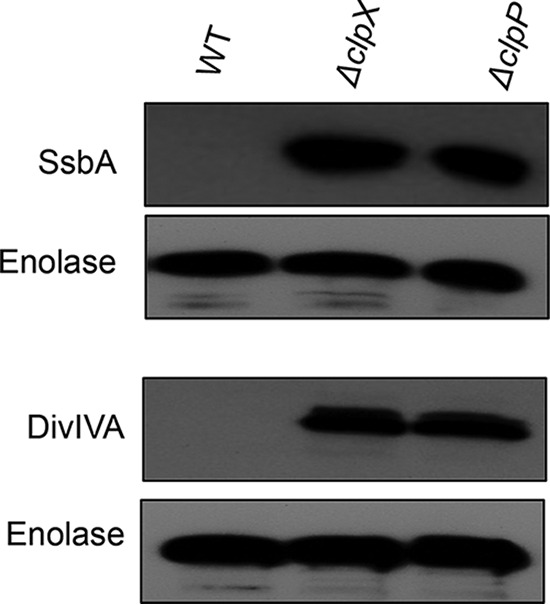
SsbA and DivIVA proteins are degraded by ClpXP in *S. mutans*. *ssbA* and the DivIVA gene were cloned in pIB190 vector in N-terminal histidine-tagged form and were transformed in the wild-type (WT) UA159 strain and its isogenic Δ*clpX* and Δ*clpP* mutant strains. After cell lysis, equal amounts of protein (~20 µg) were loaded in all lanes. The amounts of SsbA and DivIVA present in each strain were evaluated using anti-His antibody. The blots were developed with a chemiluminescent kit (ECL Plus; Thermo Scientific) by exposure to X-ray film and then photographed. Endogenous enolase was used as a loading control. Experiments were repeated at least three times, and the respective areas of a representative blot are shown.

Other than the SsrA-tagged substrates, very little is known about the identity of the substrates that are recognized by ClpX in *S. mutans*. Our proteomic study indicated that at least three proteins, AltA, DivIVA, and SsbA, could be targeted by ClpXP-mediated degradation, probably by recognizing certain motifs present in the proteins. To identify the motifs that are recognized by ClpX, we chose to study DivIVA and SsbA further, as these proteins are relatively small (31 and 18 kDa, respectively). We first cloned the genes encoding the respective proteins in a shuttle expression vector, pIB190, which expresses the proteins with an N-terminal 6× histidine (6×His) tag. These constructs were then introduced into UA159, IBSJ4 (Δ*clpX*), and IBS512 (Δ*clpP*) cells. We then measured the levels of SsbA and DivIVA proteins in these strains by Western blot assays using anti-His antibody. We also used anti-enolase antibody to ensure that equal amounts of proteins were loaded into all lanes. As shown in Fig. 1, both the DivIVA protein and the SsbA protein were barely visible in the wild-type strain, whereas they were both accumulated in the Δ*clpX*- and Δ*clpP*-deficient strains. Taken together, our results suggest that both DivIVA and SsbA are the bona fide substrates for ClpX.

### ClpXP recognizes three residues at the C terminus of the SsbA.

**FIG 2:**
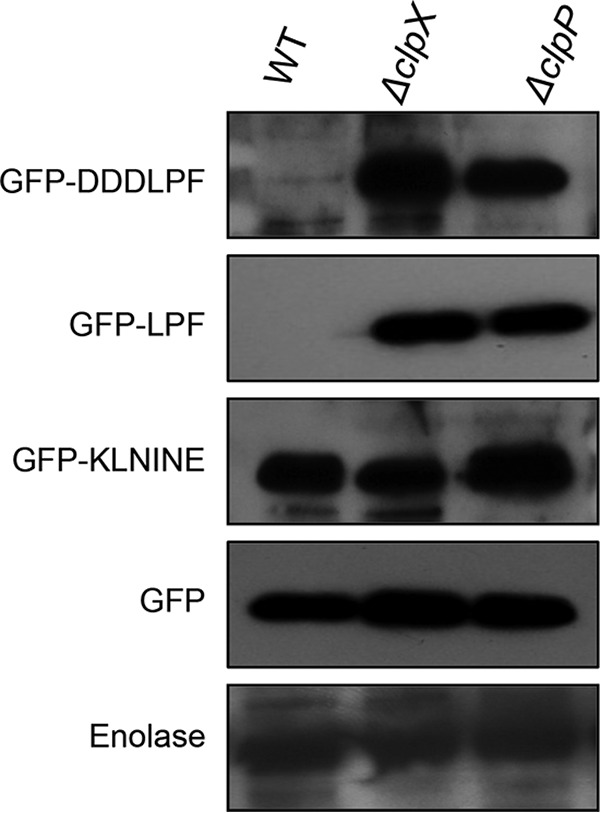
The last 3 amino acids (LPF) of SsbA are the recognition motif for ClpX in *S. mutans*. GFP with DDDLPF and LPF (last 6 and 3 amino acids of SsbA, respectively) at the C terminus was cloned in pIB190 vector with an N-terminal histidine tag for protein expression. Similarly, GFP with KLNINE (last 6 amino acids of DivIVA) at the C terminus was cloned into pIB190 for expression. The levels of GFP expressed in the cell lysate of UA159 and its isogenic Δ*clpX* and Δ*clpP* mutant strains were measured by Western blot analysis using anti-His antibody. GFP without any C-terminal tag was used as a negative control. Enolase was used as a loading control. Experiments were repeated at least twice, and a representative blot is shown.

To identify the sequences recognized by ClpXP, we focused on the C-terminal domain, since both the substrates were tagged at the N terminus with 6×His residues. Furthermore, *E. coli* ClpXP is known to recognize various C-terminal tags (16). Since the tags that are recognized by *E. coli* ClpXP are small (three to six residues in length), we attached the last six residues of the DivIVA and SsbA to the green fluorescent protein (GFP), which we had previously found to be suitable for proteolytic studies (22). Addition of the six residues (KLNINE) derived from DivIVA to GFP did not render the protein degradable by ClpXP, whereas the addition of six residues (DDDLPF) derived from SsbA made the GFP susceptible to ClpXP-mediated degradation (Fig. 2). Deletion of the last six residues from SsbA rendered the protein refractory to degradation (Fig. S2). On the other hand, when the last six residues were deleted from DivIVA, ClpXP was still able to degrade DivIVA, suggesting that last six residues are not required for ClpXP recognition (Fig. S2).

10.1128/mSphere.00287-16.5 Figure S2  *In vivo* degradation of substrates. DivIVA protein and SsbA protein in UA159 and its isogenic *ΔclpX* mutant of *S. mutans* are shown. The open reading frame of the DivIVA gene or *ssbA* without the last 6 amino acids was cloned in pIB190 vector to create an N-terminal His-tagged version. The construct was transformed into UA159 and *ΔclpX* strains. Crude lysates were prepared from the strains, and Western blotting was performed using anti-His antibody. Enolase was used as a loading control. A representative blot is shown. Download Figure S2, PDF file, 0.1 MB. Copyright © 2016 Jana et al. 2016 Jana et al. This content is distributed under the terms of the Creative Commons Attribution 4.0 International license.

**FIG 3:**
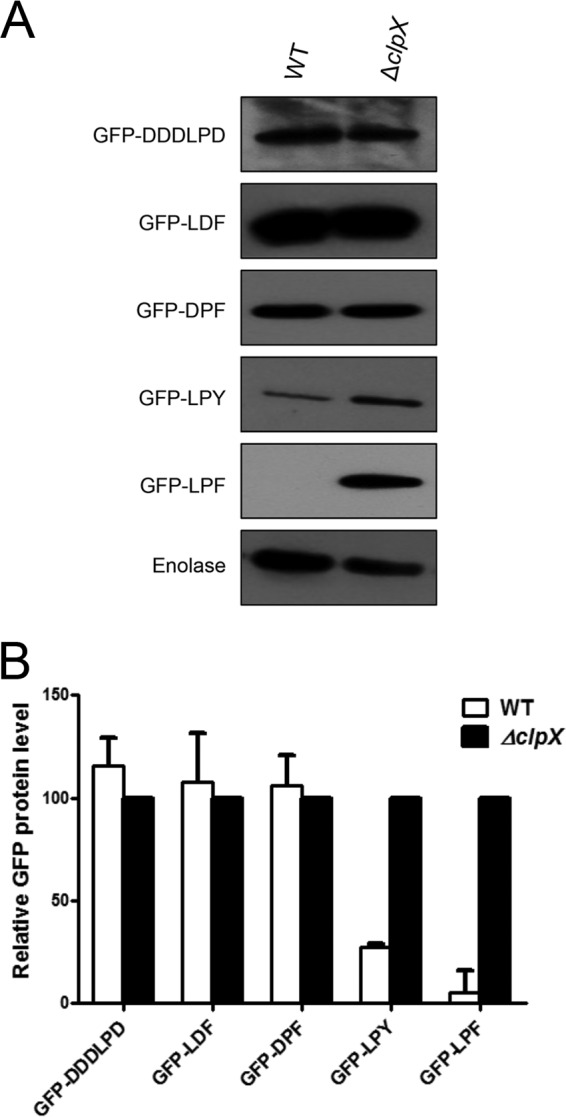
Alteration of the amino acid in the recognition motif affects ClpX recognition. (A) GFP-LPF, GFP-LDF, GFP-DPF, GFP-LPY, and GFP-DDDLPD constructs were expressed from the pIB190 vector in UA159 and its isogenic Δ*clpX* strains. Anti-His antibody was used to analyze the level of GFP present in the cell lysates. Experiments were repeated at least twice, and a representative blot is shown. (B) Relative abundances of GFP in UA159 and its isogenic Δ*clpX* mutant strains. The blot was scanned using ImageJ software, and the relative value of GFP in Δ*clpX* was set as 100.

To determine the minimum length of the motif that is recognized by *S. mutans* ClpXP, we made several sequential deletion derivatives of the six-residue tags derived from SsbA and added these tags to GFP. As shown in Fig. 2, addition of just the last three residues (LPF) to GFP rendered the protein susceptible to degradation by *S. mutans* ClpXP (please see Fig. S3). To understand the importance of each residue and its position in the motif for ClpXP recognition, we made several single amino acid substitutions in the LPF sequence. As shown in Fig. 3, it appears that for accurate ClpXP recognition, all three positions are very important. Thus, our results indicate that *S. mutans* ClpXP recognizes the C-terminal LPF motif present in the target substrate.

10.1128/mSphere.00287-16.6 Figure S3  The last 3 amino acids (LPF) of SsbA protein are the recognition sequence for *S. mutans* ClpX. (A) N-terminal His-tagged GFPs carrying a DDDLPF, DDLPF, DLPF, or LPF tag at the C-terminal end were expressed from pIB190 in the wild-type and *clpX* mutant strains. The level of GFP was measured by Western blot analysis using anti-His antibody. GFP without any tag was used as a control protein. (B) Bar diagram of relative GFP levels in the wild type and the *clpX* mutant. The relative value of GFP present in the *clpX* mutant was set as 100. Download Figure S3, PDF file, 0.1 MB. Copyright © 2016 Jana et al. 2016 Jana et al. This content is distributed under the terms of the Creative Commons Attribution 4.0 International license.

### SsbA is not degraded in a *clpX*-deficient strain.

**FIG 4:**
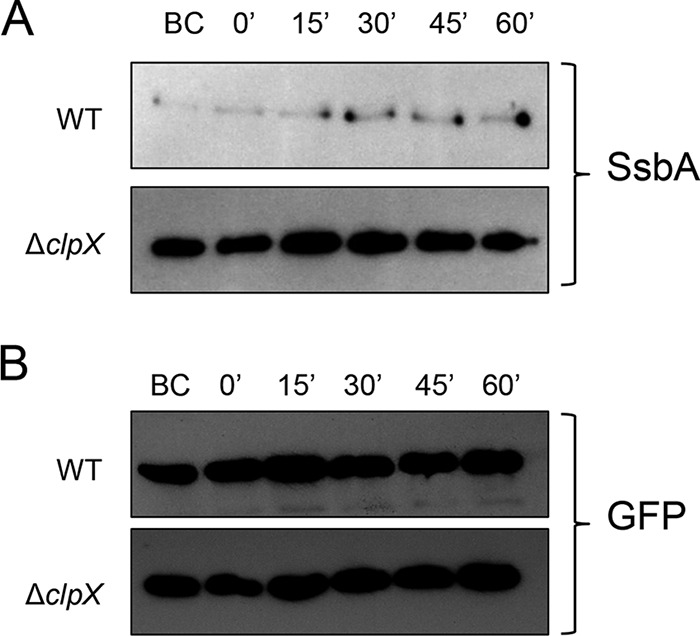
Stability of SsbA (A) and GFP (B) proteins in UA159* and ΔclpX* strains. UA159 and its isogenic *ΔclpX* strains expressing SsbA and GFPs were grown to mid-log phase (OD_600_, ~0.7). Chloramphenicol was added to stop the protein synthesis, and the culture was further incubated for up to 1 h. At different time intervals, 10 ml culture was taken out and cell lysates were prepared as described in the text. Equal amounts of proteins were loaded in all lanes. The amounts of SsbA and GFP present in cell lysates were detected by Western blot analysis using anti-His antibody. BC, samples collected just before addition of chloramphenicol.

To gain insight into the mechanism of ClpXP function, we performed a time course experiment to examine the stability of SsbA in the wild-type strain and the *clpX*-deleted strain under nonstressed conditions. As shown in Fig. 4, SsbA was rapidly degraded in the wild-type strain. In fact, we found only trace amounts of SsbA immediately before the addition of chloramphenicol and they remained the same up to the end of the time course (1 h). On the other hand, the amount of SsbA was constant in the *clpX* mutant throughout the time course of the experiment (Fig. 4A). Therefore, our results suggest that SsbA is highly unstable in *S. mutans*; however, when *clpX* was absent, SsbA was not degraded and accumulated in the cell. A similar observation was also made when GFP-LPF was used as the substrate (Fig. S4).

10.1128/mSphere.00287-16.7 Figure S4  Stability of GFP-LPF protein in the *ΔclpX* mutant. A *ΔclpX* strain expressing GFP-DDDLPF protein was grown to mid-log phase (OD_600_, ~0.7). Chloramphenicol was added to stop the protein synthesis, and the culture was further incubated for up to 1 h. At different time intervals, 10 ml culture was taken out, cell lysates were prepared, and equal amounts of proteins were loaded in all lanes. The amounts of GFP present in cell lysates were detected by Western blot analysis using anti-His antibody. A representative blot is shown. Download Figure S4, PDF file, 0.04 MB. Copyright © 2016 Jana et al. 2016 Jana et al. This content is distributed under the terms of the Creative Commons Attribution 4.0 International license.

### SsbA is not recognized by other Clp ATPases in *S. mutans*.

**FIG 5:**
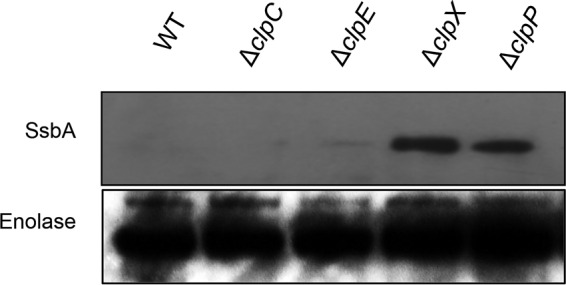
ClpC and ClpE are not responsible for SsbA degradation in *S. mutans*. The level of His-SsbA expression was measured in UA159 and its isogenic *clpC*, *clpE*, *clpX*, and *clpP* mutant strains. Enolase was used as an internal control for sample loading. Experiments were repeated at least twice, and a representative blot is shown.

We recently found that SsrA-tagged proteins in *S. mutans* are degraded by ClpCP and ClpEP, in addition to being degraded by ClpXP (23). We therefore wanted to study whether ClpC or ClpE could also recognize SsbA protein for degradation. For this, we introduced the plasmid carrying SsbA with the N-terminal histidine (His) tag into the IBSJ2 (Δ*clpC*) and IBSJ5 (Δ*clpE*) strains (25). Expression of the SsbA protein was then evaluated in these strains at the mid-logarithmic-growth phase by Western blotting. As shown in Fig. 5, neither ClpC nor ClpE recognized SsbA in *S. mutans*. Thus, SsbA is specifically targeted by ClpXP-mediated degradation and not by ClpCP or ClpEP proteolytic complexes in *S. mutans*.

### *S. mutans* ClpXP also degrades SsbB containing the LPF motif.

**FIG 6:**
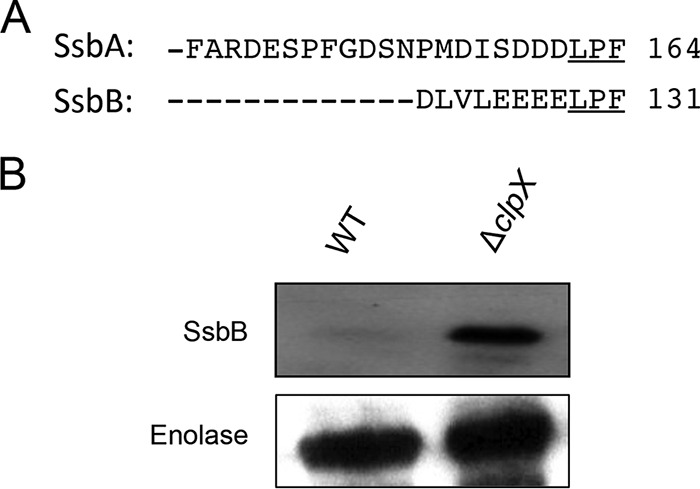
ClpX also recognizes SsbB protein in *S. mutans*. (A) Sequence alignment of the C-terminal end of SsbA and SsbB proteins. (B) Western blot analysis of SsbB in the UA159 and *ΔclpX* strains. SsbB protein was expressed from pIB190 as an N-terminal His-tagged protein. Western blot assays were done using anti-His antibody as described above. Experiments were repeated at least three times, and a representative blot is shown.

Since ClpXP recognizes the C-terminal LPF motif on the target substrates, we scanned the *S. mutans* genome for the presence of the LPF motif at the C terminus. We found only two additional proteins, namely, SsbB (SMU.1967) and a hypothetical protein encoded by SMU.394, with the LPF motif in the *S. mutans* UA159 genome. To verify whether ClpXP recognizes the SsbB protein, we cloned SMU.1967 in plasmid pIB190 so that SsbB could be expressed as N-terminal histidine-tagged protein. The construct was then introduced into the wild-type (UA159) and Δ*clpX* (IBSJ4) strains. As shown in Fig. 6, SsbB, like SsbA, was rapidly degraded in the wild-type strain, whereas SsbB was highly stable in the Δ*clpX* mutant strain. Thus, *S. mutans* ClpXP also recognizes the SsbB protein in addition to the SsbA protein.

### Recognition of the LPF motif by ClpXP is strain dependent.

**FIG 7:**
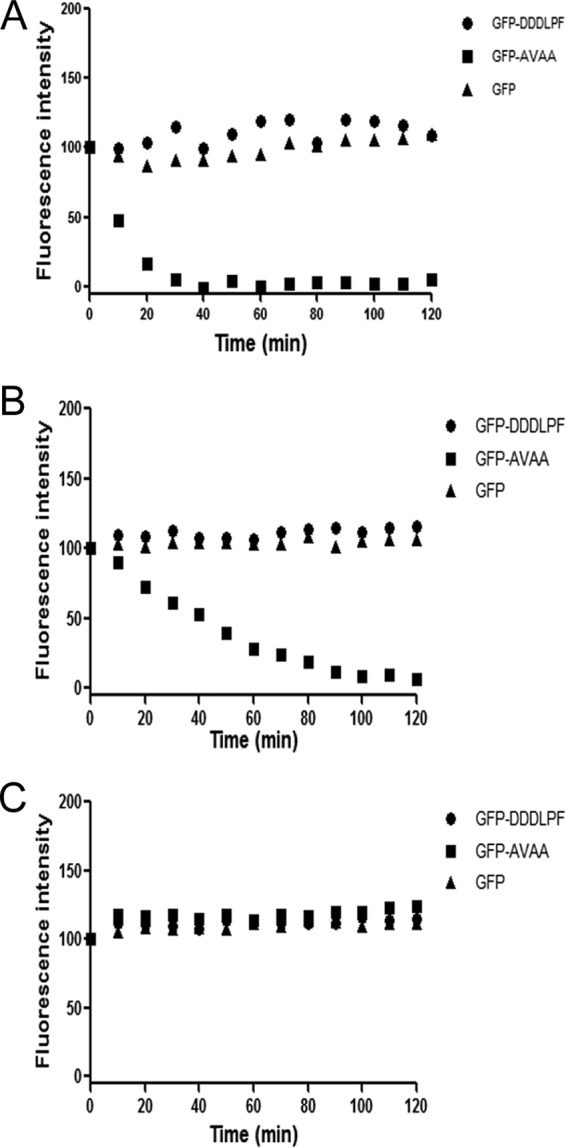
*In vitro* substrate degradation by ClpXP. Various tagged GFP substrates were incubated with preformed ClpXP complex, and fluorescence was measured at indicated time points up to 120 min and normalized against the initial value. Experiments were repeated at least three times. Panel A shows purified ClpP and ClpX proteins of *S. mutans*, whereas panel B shows ClpP of *S. mutans* and ClpX of *S. gordonii*. Panel C shows only ClpP protein. GFP-AVAA was used as a positive control. Experiments were repeated at least three times, and a representative blot is shown.

We previously showed that ClpXP could degrade GFP with the AVAA tag at the C terminus *in vitro*. To verify whether ClpXP is able to recognize the LPF motif *in vitro*, we used GFP with the LPF tag (GFP-LPF) as the substrate. We also included GFP and GPF-AVAA as negative and positive controls, respectively. As expected, ClpP alone did not show any degradation of any of the substrates (Fig. 7C) and ClpXP showed rapid degradation of GFP-AVAA (Fig. 7A). Half of the GFP-AVAA substrates were degraded at 10 min after ClpXP addition, and complete degradation was observed within 40 min (Fig. 7A and B). On the other hand, we observed no degradation of GFP-LPF even after prolonged (up to 2 h) incubation. This result suggests that either the *in vitro* condition is not optimal for the recognition of the LPF motif or an accessory factor such as an adaptor protein is needed for the recognition.

**FIG 8:**
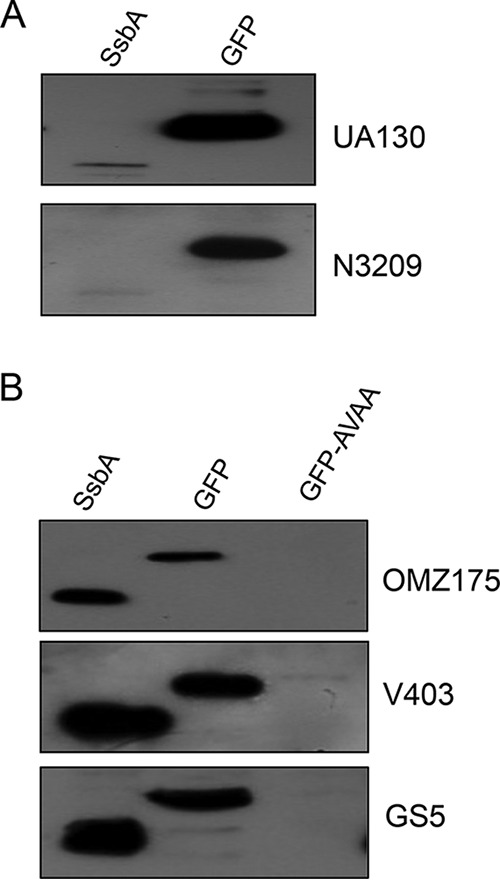
Recognition of the LPF motif in various *S. mutans* isolates. Panel A shows the strains that successfully recognized the LPF motif for degradation, whereas panel B shows the strains that failed to recognize the motif. GFP construct was used as a negative control, whereas GFP-AVAA was used as a positive control to demonstrate that ClpXP is functional in those strains that do not degrade LPF. Experiments were repeated at least twice, and a representative blot is shown.

The *S. mutans* genome is composed of roughly 1,500 core genes and about 4,000 pan-genes (26). While the *clpX* and *clpP* genes are considered part of the core genome, there is a pronounced strain-to-strain variability present among various *S. mutans* strains. Thus, we wanted to evaluate whether recognition of the LPF motif by ClpXP in *S. mutans* is a universal phenomenon or is strain dependent. For this, we included five additional strains (GS-5, N3209, OMZ175, UA130, and V403); the complete genome sequences for all, except UA130, are in the public domain. These strains were transformed with SsbA and GFP constructs and were evaluated for ClpXP-mediated degradation. As shown in Fig. 8A, LPF was degraded only in N3209 and UA130. On the other hand, as shown in Fig. 8B, the LPF motif was not recognized in strains GS-5, OMZ175, and V403. To confirm that ClpXP is functional in the latter strains, we introduced GFP-AVAA in GS-5, OMZ175 and V403 strains. As shown in Fig. 8B, GFP-AVAA was rapidly degraded in all three strains, indicating that ClpXP is functional in these strains. Together, these data suggest that the LPF motif is recognized by some but not all *S. mutans* strains.

### The LPF motif is also not recognized by ClpXP in other bacteria.

**FIG 9:**
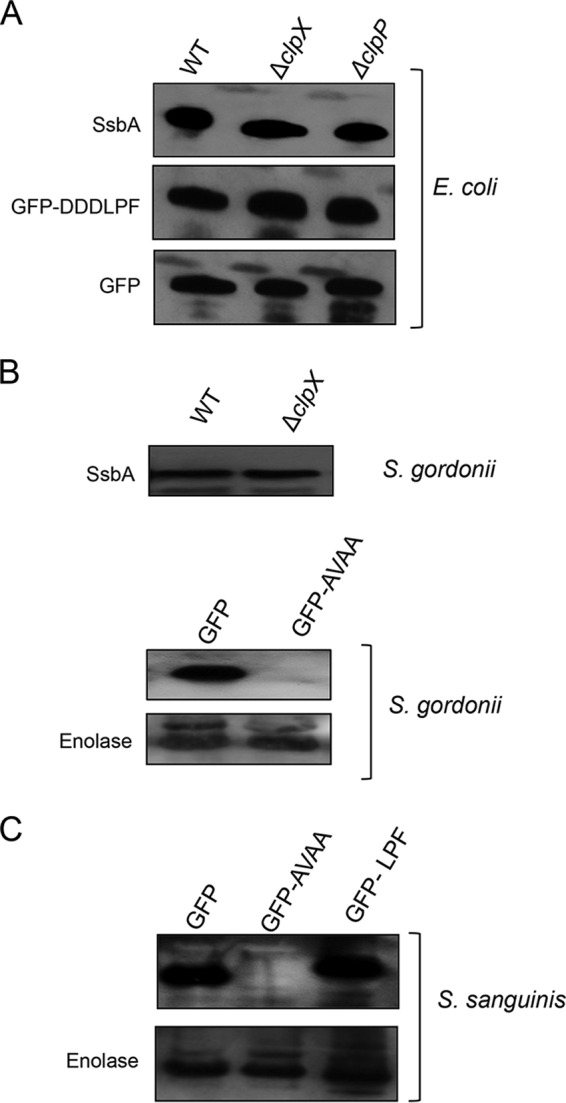
The LPF motif is not recognized in *E. coli* and streptococci. (A) Plasmids containing SsbA (LPF), GFP, or GFP-DDDLPF were transformed into *E. coli* JW0427 and its isogenic *ΔclpX* and *ΔclpP* strains (Table 1). The levels of His-SsbA, GFP-DDDLPF, and GFP expression were analyzed by Western blotting using anti-His antibody. (B) Recognition of the LPF motif in *S. gordonii*. His-SsbA was also expressed in *S. gordonii* DL-1 and its isogenic *clpX* mutant strains. The level of SsbA expression was measured by Western blot analysis using anti-His antibody. The GFP-AVAA construct was used as a positive control to demonstrate that ClpXP is functional in DL-1. Experiments were repeated at least three times, and a representative blot is shown. Enolase was used as a loading control only for the lower panel. (C) Recognition of the LPF motif in *S. sanguinis*. GFP, GFP-LPF, and GFP-AVAA constructs were introduced into *S. sanguinis* SK-36. Protein expression was measured using anti-GFP antibody. Enolase was used as a loading control. Experiments were repeated at least twice, and a representative blot is shown.

To determine whether ClpXP from other organisms recognizes SsbA or the last six residues of SsbA, we first selected *E. coli*. We introduced either an SsbA protein-encoding plasmid or GFP-DDDLPF into wild-type *E. coli* as well as *clpX*- and *clpP*-deficient mutants of *E. coli* and measured the amounts of SsbA or GFP accumulation in the cell. As shown in Fig. 9A, we observed that GFP, GFP-DDDLPF, and SsbA accumulated in comparable amounts in all the strains, suggesting that neither SsbA nor the GFP-DDDLPF tag was recognized by *E. coli* ClpXP.

**TABLE 1:** Strains and plasmids used in the study

Strain or plasmid	Description^a^	Reference or source
Strains		
* S. mutans* UA159	Wild type, serotype c	17
* S. mutans* IBS512	UA159 derivative Δ*clpP*	40
* S. mutans* IBSJ2	UA159 derivative Δ*clpC*	46
* S. mutans* IBSJ4	UA159 derivative Δ*clpX*	46
* S. mutans* IBSJ5	UA159 derivative Δ*clpE*	46
* S. gordonii* DL-1	Wild type	
* S. gordonii* IBSP1	*clpX* is disrupted by *lox*-Kan in DL-1, Kan^r^	This study
* S. sanguinis*	SK-36, wild type	
* E. coli* K-12	Wild type	47
* E. coli* JW0427-1	K-12 derivative Δ*clpP*	47
* E. coli* JW0427-1	K-12 derivative Δ*clpX*	47
		
Plasmids		
pGEM-T Easy	Commercial TA cloning vector, Amp^r^	Promega
pCrePA	Thermosensitive plasmid with Cre recombinase, Em^r^	48
pIB190	A shuttle vector for protein expression in *S. mutans*, Em^r^	38
pIBP1	pIB190 with His-SsbA, Em^r^	This study
pIBP92	pIB190 with His-SsbA Δ6, Em^r^	This study
pIBP6	pIB190 with His-GFP, Em^r^	This study
pIBP7	pIB190 with His-GFP-DDDLPF, Em^r^	This study
pIBP56	pIB190 with His-GFP-DDLPF, Em^r^	This study
pIBP57	pIB190 with His-GFP-DLPF, Em^r^	This study
pIBP58	pIB190 with His-GFP-LPF, Em^r^	This study
pIBP59	pIB190 with His-GFP-DDDLPD, Em^r^	This study
pIBP68	pIB190 with His-GFP-LDF, Em^r^	This study
pIBP69	pIB190 with His-GFP-DPF, Em^r^	This study
pIBP70	pIB190 with His-GFP-LPY, Em^r^	This study
pIBP72	pIB190 with SsbB, Em^r^	This study
pIBP64	pGEM-T Easy with *S. gordonii clpX*, Amp^r^	This study
pIBP73	pGEM-T Easy with *S. gordonii clpX* disrupted by *lox*-Kan cassette, Amp^r^, Kan^r^	This study
pIBP2	pIB190 with His-DivIVA, Em^r^	This study
pIBP5	pIB190 with His-GFP-KLININE, Em^r^	This study
pIBP74	pIB190 with His-DivIVAΔ6, Em^r^	This study
pIBP91	pIB190 with His-GFP-SsrA-AVAA, Em^r^	This study
pIBP12	pETDuet vector with His-GFP, Amp^r^	This study
pIBP13	pETDuet vector with His-GFP-DDDLPF, Amp^r^	This study
pIBP86	pET3a with *S. mutans* ClpP	This study
pIBJ27	pASK43+ with His-GFP-AVAA, Amp^r^	22
pIBJ36	pASK43+ with *S. mutans* ClpX, Amp^r^	22

a Kan^r^, kanamycin resistance; Amp^r^, ampicillin resistance; Em^r^, erythromycin resistance.

We then extended the study to *Streptococcus gordonii*, also an oral bacterium. To this end, we wanted to construct both *clpP*- and *clpX*-deficient derivatives of *S. gordonii*. After repeated attempts, we were unable to generate a *clpP*-deficient *S. gordonii*. However, we were successful in generating a *clpX*-deficient derivative. We introduced the SsbA-encoding plasmid into both the wild-type and the *clpX*-deficient *S. gordonii* strains and measured the SsbA accumulation by Western blotting. As shown in Fig. 9B, we did not observe any significant difference in the SsbA amounts in these strains, suggesting that *S. gordonii* ClpXP does not recognize *S. mutans* SsbA and hence the LPF motif. Similarly, we found that *S. sangunis* ClpXP does not degrade the LFP motif (Fig. 9C). Among the species tested, our data clearly suggest that the LPF motif is selectively recognized by *S. mutans* ClpXP.

## DISCUSSION

Regulated proteolysis is an essential physiological process responsible for cellular protein homeostasis and protein quality control in bacteria. Key to this important process is the involvement of the ClpXP proteolytic complex. As is the case in other well-studied bacteria such as *E. coli* and *Bacillus subtilis*, ClpXP is the major proteolytic complex involved in protein quality control in *S. mutans* (19, 22, 27). As expected from its role in regulated proteolysis, inactivation of either *clpP* or *clpX* leads to pleotropic effects, including defects in biofilm formation, bacteriocin production, and competence development (19, 27). While we know the importance of ClpXP in cellular physiology, the substrates recognized by this proteolytic complex are largely unknown. In this study, we used two-dimensional polyacrylamide gel electrophoresis (2D-PAGE) to identify putative substrates that are recognized by ClpX. We found both up- and downregulated proteins by proteomic analysis. While the exact reason for the presence of downregulated proteins in the ClpX-deficient mutant is not known, we speculated that some of the upregulated proteins are the substrates for ClpX. Surprisingly, among the 17 spots that we identified by liquid chromatography-mass spectrometry (LC-MS), none were identified by our previous proteomic study of ClpP-dependent expression (19). The probable reasons could be attributable to differences in growth conditions, incomplete identification of the protein spots, and redundant activity of some of the Clp ATPases. Nevertheless, using tagged proteins in an *in vivo* degradation assay, we confirmed the identities of two proteins that are bona fide ClpX substrates.

ClpXP-mediated substrate degradation often requires short peptide motifs that are recognized by the protease complex (1). Among these motifs, the best-studied one is encoded by an SsrA tag, which generally adds an ~11-residue peptide with either AVAA or ALAA at the C terminus. The SsrA tag is highly conserved in bacteria and is recognized by ClpX in many bacterial species, including *S. mutans*. Although several ClpX substrates have been identified in other bacteria, including *B. subtilis*, the short degradation motifs (three residues) that are recognized by ClpX have been documented only in *E. coli* (15, 28). In that bacterium, ClpX can recognize five types of motifs: three located at the N terminus and two at the C terminus of the target proteins (15). In this study, we identified a three-residue-long C-terminal motif (LPF) that is recognized by *S. mutans* ClpX. Interestingly, it was previously reported that ClpC in *B. subtilis* also recognizes a short motif (LCN) located at the C terminus of SpoIIB protein (29); however, no such short motif is reported for ClpX ATPase. To the best of our knowledge, LPF is the first such short motif identified in any organism other than *E. coli* that is solely recognized by ClpX ATPase.

GPF is a folded protein whose last three residues are LYK and is not degraded by ClpXP. We found that addition of LPF to GFP leads to complete degradation of the protein and that three residues are sufficient for the recognition and unfolding by ClpX ATPase. When any of these residues were replaced with other residues, ClpX was unable to recognize the GFP. Among the replacements that we tested, the only substitution in the LPF motif that was tolerated by ClpX was the last residue, phenylalanine, replaced with tyrosine. Since these 2 amino acids share similar structural physiochemical properties, it was not surprising that ClpX could also recognize LPY. The mere presence of a phenylalanine or tyrosine residue along with proline (PF or PY) at the C terminus of a protein is also not sufficient for the recognition. However, we have not extensively and systematically tested all the residues for ClpXP recognition. We also found that the LPF motif is not recognized by any other Clp ATPases (Fig. 3), suggesting that LPF is specific for ClpXP.

In the *S. mutans* core genome, only three proteins contain the LPF motif and one protein contains the LPY motif. We tested two of the LPF-containing proteins, SsbA and SsbB; both are single-strand DNA binding proteins. These two proteins are highly similar to the SsbA and SsbB proteins from *Streptococcus pneumoniae*. The SsbA protein is similar in size to the well-characterized SSB protein from *E. coli* (SsbEc). The SsbB protein, in contrast, is a smaller protein that is specifically induced during natural transformation in *S. pneumoniae* and has no counterpart in *E. coli* (30); the amount of SsbB is about 20 times more than the amount of SsbA during competence in *S. pneumoniae* (30). Even after overexpression, we could barely detect the SsbA (or SsbB) protein in the wild-type *S. mutans* strain, indicating rapid degradation of the proteins. It is not clear why these key proteins are rapidly degraded in *S. mutans*, but the reason could be that the proteins are required to maintain stringency of competence development. Furthermore, since these proteins are not degraded in all *S. mutans* strains, how the competence is regulated in these strains remains unclear. We speculate that, in strains where SsbA/B and DivIVA are rapidly degraded, either additional regulators are involved that help to modulate the expression of ClpXP or some key protein (adaptor; see below) controls the function of the ClpXP complex. During competence development, the abundance of these regulators or of the adaptors is altered and the Ssb and DivIVA proteins become transiently available.

The other LPF-containing protein is SMU.394, which encodes a nucleoid-associated protein of the EbfC family (31). The role of this protein in *S. mutans* is currently unknown, but its homolog in *Borrelia burgdorferi* is involved in virulence gene regulation (31). A search for LPF-containing protein in the *S. mutans* pan-genome also identified another small (~25-kDa) protein of the Abi C family (GenBank accession number AFM81543). Abi C family proteins are involved in abortive phage infections (32). The Abi C protein that we found is present in about 25 *S. mutans* strains, including GS-5, but the exact function of this protein is currently unknown. On the other hand, the LPY motif-containing protein that we identified is an ABC transporter protein (SMU.459) that might be involved in amino acid (cysteine) transport and is well conserved across the *S. mutans* strains.

Recently (in 2010), Niu and colleagues identified another motif, VSA, which is present internally in the IrvR repressor (24). Upon autocatalytic cleavage of IrvR, the VSA motif is amenable to degradation. However, those authors found that simply adding the VSA motif does not render the substrate sensitive to ClpXP-mediated degradation; the additional three residues are required for complete degradation (24). Furthermore, ClpC also recognizes this short six-residue motif that is not specific for ClpX recognition. We have found that DivIVA is also a bona fide substrate for ClpX recognition. However, in this case, ClpX appears to recognize some motifs that are not located at the C terminus. This is because deletion of the last six residues (KLNINE) from DivIVA did not protect the substrate from ClpXP-mediated degradation. Furthermore, addition of the KLNINE motif to GFP did not make the protein sensitive to ClpXP-mediated degradation. DivIVA is a conserved protein in Gram-positive bacteria that localizes at the pole and division sites (33). In *S. pneumoniae*, DivIVA is also phosphorylated by a eukaryotic-like Ser/Thr kinase (StkP) (34). Protein phosphorylation often plays a role in degradation. For example, autophosphorylation of a eukaryotic-like tyrosine kinase in *E. coli* stabilizes the protein and protects it from degradation (35). In *B. subtilis*, protein phosphorylation leads to degradation of certain proteins by Clp proteolytic complexes (36). At present, we do not know whether protein phosphorylation plays a role in the degradation of DivIVA by ClpXP in *S. mutans*. However, we are in the process of evaluating the role of Ser/Thr phosphorylation in substrate degradation by ClpXP. It is also not clear how the level of DivIVA is modulated in the cell during cell division, since it is an important protein required for cell division.

We found that other streptococci such as *S. sanguinis* and *S. gordonii* do not recognize the LPF tag. Surprisingly, we also found that the LPF tag is recognized by a subset of *S. mutans* strains but not by all isolates. While the exact reason for this strain-specific phenomenon is under investigation, we can speculate with respect to two different scenarios. First, it is possible that the LPF tag is not directly recognized by the ClpXP complex and that the tag is instead brought to the complex by an adaptor protein. To identify the putative adaptor protein, we compared the genome sequences of two strains that recognize the tag (UA159 and N3209) with the genome sequences of the two strains that do not (GS-5 and OMZ175). We were unable to find any strain-specific protein that is present in UA159 and N3209 but absent in GS-5 and OMZ175. The absence of a strain-specific protein does not rule out the possibility of involvement of an adaptor protein for several reasons. For example, the adaptor protein may be encoded by GS-5 and OMZ175 but may not be expressed in the strains. Alternatively, the adaptor may be encoded and expressed but may carry some mutations that render the protein nonfunctional. Another possibility is the presence of an inhibitory protein in the strains that do not recognize the tag because the protein sequesters the LPF tag such that ClpXP is unable to access the tag. In fact, the charged C-terminal tail of Ssb proteins is known to recruit at least 12 proteins that are involved in chromosome stability (37). Interestingly, our *in vitro* assay using purified ClpXP was unable to degrade the LPF-tagged substrates. It is also possible that the substrate used for *in vitro* studies was isolated from *E. coli* and therefore was folded in such a way that ClpXP could not recognize the LPF tag. On the other hand, it is also possible that the adaptor protein was absent in the *in vitro* reactions. We attempted to supplement the* in vitro* assay with crude lysate prepared from the stationary-phase UA159 culture but were unable to observe LPF degradation. This negative result suggests, among many other possibilities, either that the adaptor protein was not very stable or that it was not present in high abundance. An extensive in-depth analysis involving both genetics and biochemical approaches is necessary to identify the adaptor protein required for ClpXP activity in *S. mutans*.

## MATERIALS AND METHODS

### Bacterial growth conditions.

*E. coli* strains were grown in Luria-Bertani (LB) medium supplemented with erythromycin (Er; 500 µg/ml), ampicillin (Amp; 100 µg/ml), and kanamycin (Kan; 50 µg/ml) (whenever required). *S. mutans* (UA159 and its derivatives) strains were routinely grown in Todd-Hewitt medium supplemented with 0.2% yeast extract (THY medium). Whenever necessary, 5 µg/ml erythromycin or 400 µg/ml kanamycin was added to the THY medium.

### Plasmid construction.

To express SsbA and SsbB in *S. mutans* as N-terminal histidine-tagged variants, plasmids pIBP1 and pIBP72, respectively, were constructed by cloning *ssbA* and *ssbB* (using UA159 genomic DNA as a template) in the pIB190 vector (38). To express GFP with various tags at the C-terminal end, pIBP6 (GFP alone), pIBP7 (GFP-DDDLPF), pIBP57 (GFP-DDLPF), pIBP58 (GFP-DLPF), pIBP59 (GFP-LPF), and pIBP5 (GFP-KLNINE) were generated. Plasmids pIBP68, pIBP69, and pIBP70 were constructed by site-directed mutagenesis using pIBP59 as a template. To check the stability of DivIVA and DivIVAΔ6 (without the last six residues), pIBP2 and pIBP72, respectively, were constructed using pIB190. All pIB190-specific derivatives contained a His tag (6×) at the N-terminal end. All plasmids were transformed in various *S. mutans* strains by natural transformation. Transformants were verified by PCR using vector (pIB190)-specific primers (pIB190F and pIB190R). Primers used for constructing these plasmids are listed in Table S1 in the supplemental material.

10.1128/mSphere.00287-16.1 Table S1  Oligonucleotides used in the study. Download Table S1, PDF file, 0.1 MB. Copyright © 2016 Jana et al. 2016 Jana et al. This content is distributed under the terms of the Creative Commons Attribution 4.0 International license.

10.1128/mSphere.00287-16.2 Table S2  Differential expression of proteins in UA159 versus the isogenic *ΔclpX* strain. Download Table S2, PDF file, 0.1 MB. Copyright © 2016 Jana et al. 2016 Jana et al. This content is distributed under the terms of the Creative Commons Attribution 4.0 International license.

10.1128/mSphere.00287-16.3 Table S3  Differential expression of proteins identified by mass spectroscopy. Download Table S3, PDF file, 0.1 MB. Copyright © 2016 Jana et al. 2016 Jana et al. This content is distributed under the terms of the Creative Commons Attribution 4.0 International license.

### Construction of the *clpX* deletion in *Streptococcus gordonii*.

Deletion of *clpX* in *S. gordonii* was performed using a Cre-*loxP* protocol as described previously (39, 40). Briefly, the *S. gordonii clpX* gene fragment was cloned in pGEM-T Easy vector. Using the inverse PCR method, an internal region of the *clpX* gene was deleted and a kanamycin resistance cassette (Kan^r^) with modified *loxP* sites (*lox*71-Kan-*lox*66) was inserted within the deleted gene to generate plasmid pIBP73. Using pIBP73, the *clpX* gene disrupted by the Kan^r^ cassette was amplified with primers (SGordClpXF and SGordClpXR) and introduced into *S. gordonii* by natural transformation. The transformants were selected on THY plates containing kanamycin, and the disruption of the *clpX* gene was verified by PCR.

### Two-dimensional gel electrophoresis (2-DGE) and spot identification.

*S. mutans* strains (UA159 and IBSJ2) were grown to the mid-exponential-growth phase, and the cells were harvested by centrifugation. The cell pellets were lysed in 300 μl each of osmotic lysis buffer containing 10× nuclease stock, phosphatase inhibitor stocks (I and II), and 100 mg of washed glass beads as described by Jazwinski (41). The samples were subjected to vortex mixing (5 min), sonicated (5 min), and subjected to vortex mixing again. Protein concentrations of the supernatants were determined using the bicinchoninic acid (BCA) assay. The samples were adjusted to a concentration of 1 mg/ml in urea sample buffer before loading was performed (42). 2-DGE was performed according to the carrier ampholine method of isoelectric focusing (IEF) with tropomyosin (molecular weight [MW] of 33,000 and pI 5.2) as an internal standard (42). A 10% sodium dodecyl sulfate–polyacrylamide gel electrophoresis (SDS-PAGE) procedure was carried out as the second dimension, and staining was performed with a special silver stain that is compatible with mass spectroscopy. Two independent samples were run for each strain. The differences among the spots were calculated from spot percentages (density of the individual spots divided by total density of all measured spots). Spots showing at least a 1.7-fold difference between the strains with a *P* value of ≤0.05 (Student’s *t* test) were extracted, and their identity was determined by electrospray ionization (ESI) mass spectrometry. The sample preparations and the 2-DGE were carried out at Kendrick Laboratories.

### Protein extraction and Western blot analysis.

For streptococcal protein extraction, a culture that had been grown overnight was subcultured in fresh THY medium with a 5% inoculum and was allowed to grow to late log phase (approximate optical density at 600 nm [~OD_600_], 0.7). Cultures (50 ml) were harvested by centrifugation, resuspended in 1 ml of buffer A (HEPES-NaOH [pH 7.5], 300 mM NaCl, 5% glycerol), and lysed by bead beating. The suspension was centrifuged, the supernatant (500 µl) was collected, and the total protein concentration was measured by the Bradford method (43). Equal amounts of protein (~20 µg) were loaded in all lanes and subjected to SDS-PAGE. The gels were electroblotted on a polyvinylidene difluoride (PVDF) membrane. Western blotting was performed using a standard protocol. Mouse anti-His antibody (Qiagen) or mouse anti-GFP antibody (Sigma) was used as the primary antibody and goat anti-mouse IgG peroxidase (Sigma) as a secondary antibody. Blots were developed using ECL Plus reagent (Thermo Scientific). Enolase was used as a loading control, and the same membrane was used to measure enolase levels. For this, the PVDF membrane was stripped with stripping buffer (Tris-buffered saline containing 2% SDS and 100 mM β-mercaptoethanol) and was reprobed with anti-enolase antibody. To isolate total protein from *E. coli*, a saturated culture that had been grown overnight was inoculated in LB and grown to mid-log phase (OD_600_, 0.5). Cultures (10 ml) were centrifuged, and cell pellets were lysed by sonication. Equal amounts of protein were loaded in all lanes for SDS-PAGE, and Western blotting was performed as described above.

### Time course assay for protein stability.

To measure the stability of SsbA and GFPs in *S. mutans*, UA159 and *clpX* mutant strains carrying either pIBP1 or pIBP6 were grown overnight and subcultured into fresh THY medium at 37°C with appropriate antibiotics. When the cultures reached the late-log phase (~OD_600_, 0.7), chloramphenicol (20 µg/ml) was added to inhibit protein synthesis. At different time intervals, 10-ml aliquots were recovered for total cellular lysate preparation and Western blot analyses.

### Protein purification for *in vitro* studies.

To purify native ClpP, the open reading frame was cloned in the pET3a vector. Protein expression was induced in the *E. coli* Bl21(DE3) strain using 1 mM IPTG (isopropyl-β-d-thiogalactopyranoside). After induction, the cells pellets were lysed in buffer B (25 mM HEPES-NaOH [pH 7.5], 250 mM NaCl, 5% glycerol) by sonication. Clarified cell lysate was loaded onto an SP HQ column (GE Healthcare) and subjected to anion exchange chromatography. The protein was eluted using a salt gradient of 250 to 1,000 mM NaCl, and the purity of the recovered protein was assessed by SDS-PAGE. *S. mutans* ClpX was purified as described by Tao and Biswas (22).

To purify GFP and GFP-DDDLPF, pIBP12 and pIBP13 were constructed using pETDuet1 expression vector. Protein expression was induced in the *E. coli* Bl21(DE3) strain by the use of 1 mM IPTG. Recombinant proteins were purified using nickel-nitrilotriacetic acid (Ni-NTA) (Novagen) affinity chromatography and dialyzed against buffer C (25 mM HEPES/KOH [pH 7.5], 200 mM KCl, 10% glycerol). The purity of the proteins was assessed by SDS-PAGE. GFP-AVAA was purified as described by Tao and Biswas (22).

### *In vitro* degradation assay.

*In vitro* degradation assays were performed as described previously (44, 45). Briefly, 0.3 μM His-ClpX, 0.8 μM ClpP-His, and 0.1 μM substrate GFPs were added to a 100-μl reaction system. The reactions were carried out at 25°C for 120 min in a buffer containing 25 mM HEPES-NaOH (pH 7.6), 5 mM MgCl_2_, 200 mM KCl, 0.032% NP-40, 10% glycerol, and an ATP regeneration system (4 mM ATP, 16 mM creatine phosphate, 0.032 mg/ml creatine kinase). Fluorescent signals were detected with a Synergy/H1 microplate reader (BioTek).
