# Supplementary figures and images for: Strain-Dependent Recognition of a Unique Degradation Motif by ClpXP in Streptococcus mutans
Source: mSphere. 2016 Dec 7;1(6):e00287-16. doi: 10.1128/mSphere.00287-16 (PMC5143411; doi:10.1128/mSphere.00287-16)

A

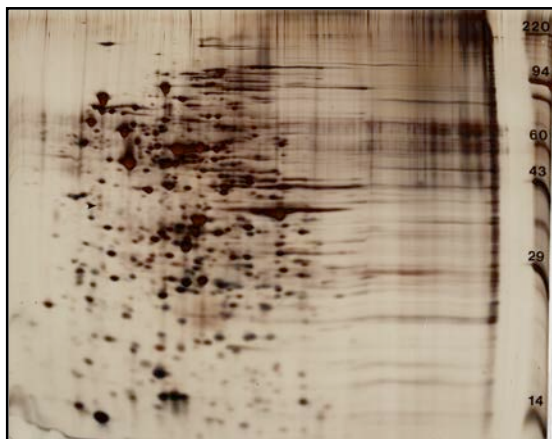

B

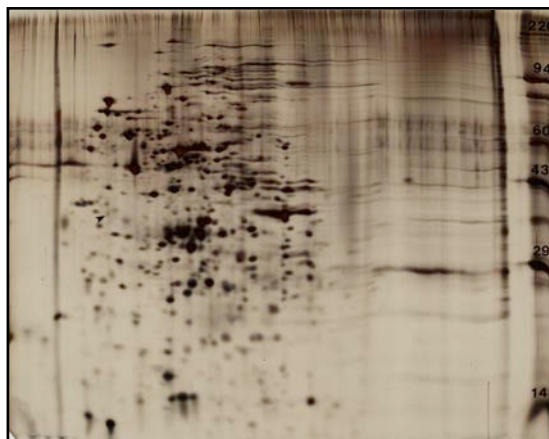

Figure S1

Supplement: Figure S1 [file sph006162201sf1.pdf]

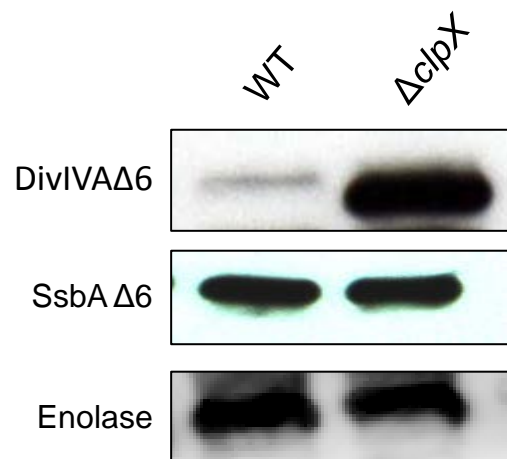

Figure S2

Supplement: Figure S2 [file sph006162201sf2.pdf]

**A**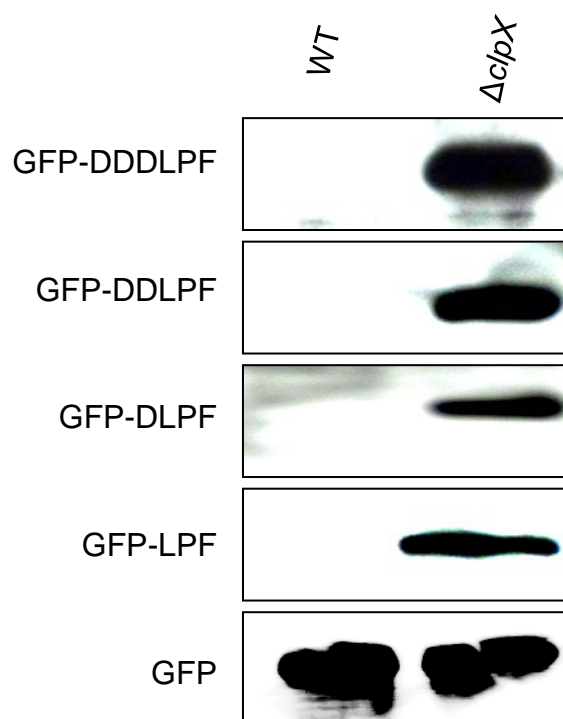**B**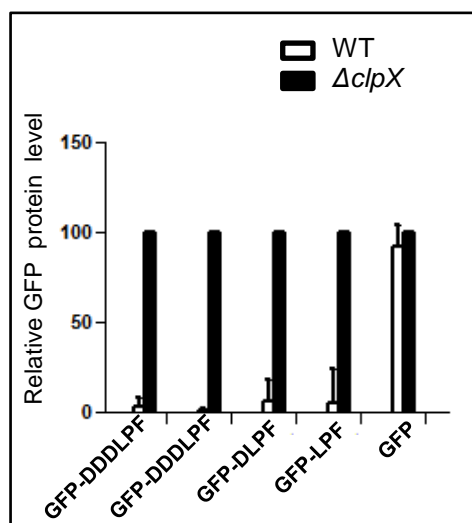

Figure S3

Supplement: Figure S3 [file sph006162201sf3.pdf]

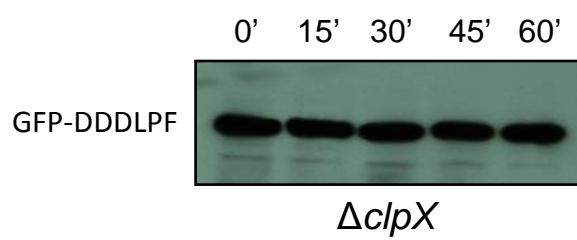

Figure S4

Supplement: Figure S4 [file sph006162201sf4.pdf]
