# Supplementary material for: Strain-Dependent Recognition of a Unique Degradation Motif by ClpXP in Streptococcus mutans
Source: mSphere. 2016 Dec 7;1(6):e00287-16. doi: 10.1128/mSphere.00287-16 (PMC5143411; doi:10.1128/mSphere.00287-16)
Supplement: Table S1 [file sph006162201st1.pdf]

Table S1: Oligonucleotides used in the study

Name	Sequence (5'-3')	Purpose
pIB190F	GGC TAG CAG AGG ATC GC	Verification of pIB190 derivatives
pIB190B	GAG GAA GCG GAA GAG CG	Verification of pIB190 derivatives
ssb his-pIB190F	AATGAATTCATGATTAATAATGTAGTACTAG	Cloning of *ssbA* in pIB190
ssb hispIB190B	ACTGGATCCTTAAAACGGAAGATCATCGTC	Cloning of *ssbA* in pIB190
divhispIB190F	CTTGGATCCATGGCAATTACAGCACTTG	cloning of *divIVA* in pIB190
divhispIB190B	ACTGCTGCAGTTATTCGTTAATATTTAATTTAAAAG	cloning of *divIVA* in pIB190
DivIVA6aaDB	GATCGTCGACTTAAAAAGTCTGTGTTTCATTC	cloning of *divIVA* without last 6 amino acids in pIB190
GFP-F190	GGATCC ATG AGT AAA GGA GAA GAA CTT	Cloning of GFP in pIB190
GFP BACWARD 190	TTA TTT GTA TAG TTC ATC CAT G	Cloning of GFP in pIB190
GFP-B-SSB190	TTA AAA CGG AAG ATC ATC GTC TTT GTA TAG TTC ATC CAT GCC	Cloning of GFP+DDDLPF construct
GFP-B-DIV	TTA TTC GTT AAT ATT TAA TTT TTT GTA TAG TTC ATC CAT GCC	Cloning of GFP+KLNINE construct
GFP-B-SSB1905AA	TTA AAA CGG AAG ATC ATC TTT GTA TAG TTC ATC CATGCC	Cloning of GFP+DDDLPF construct
GFP-B-SSB1904AA	TTA AAA CGG AAG ATC TTT GTA TAG TTC ATC CAT GCC	Cloning of GFP+DLPF construct
GFP-B-SSB1903AA	TTA AAA CGG AAG TTT GTA TAG TTC ATC CAT GCC	Cloning of GFP+LPF construct
GFP-B-SSB190mutant	TTA GTC CGG AAG ATC ATC GTC TTT GTA TAG TTC ATC CAT GCC	Cloning of GFP+DDDLPD construct
GFP-petF	GGATCC G ATG AGT AAA GGA GAA GAA CTT	Purification of GFP or GFP+DDDLPF
LPF/LPYf	TATACAAACTTCCGTATTAAAATCACTAGTG	Mutagenesis from GFP-LPF to GFP-LPY
LPF/LPYb	GTGATTTTAATACGGAAGTTTGTATAGTTC	Mutagenesis from GFP-LPF to GFP-LPY
LPF/LDFf	TATACAAACTTGACTTTTAAAATCACTAGTG	Mutagenesis from GFP-LPF to GFP-LDF
LPF/LDFb	GTGATTTTAAAAGTCAAGTTTGTATAGTTC	Mutagenesis from GFP-LPF to GFP-LDF
LPF/DPFf	TATACAAAGATCCGTTTTAAAATCACTAGTG	Mutagenesis from GFP-LPF to GFP-DPF
LPF/DPFb	GTGATTTTAAAACGGATCTTTGTATAGTTC	Mutagenesis from GFP-LPF to GFP-DPF
SSB1967F	AATGAATTCATGTATAATAAAGTTATTTTGATTG	Cloning of *ssbB* in pIB190
SSB1967B	ACT GGATCC TTAAAAAGGGAGTTCCTCCTC	Cloning of *ssbB* in pIB190
SGordClpXF	ATG CCT ACA AAT CGT AATG	Verification of *clpX* gene in *S. gordonii*
SGordClpXB	TCA AGC AGT CTC TAA AAT TG	Verification of *clpX* gene in *S. gordonii*
SGClpXDF	ACTG CTC GAG GAT GAA AAC GAG TCC TAC	Deletion of *clpX* gene in *S. gordonii*
SGClpXDB	ACTG GGA TCC GTC TTC TCC GAC ATA ACC	Deletion of *clpX* gene in *S. gordonii*
4Qlox71-Km-F-Bam	ATTGGATCCTTAGGATCCCGATAACTTCGTATAATGTA TGCTATACGAAGTTATGAGGATGAAGAGGATGAGGAGGC AG	Deletion of *clpX* gene in *S. gordonii*
4Qlox66-Km-R-Bam	ATTGGATCCTTAGGATCCCGATAACTTCGTATAGCA TACATTATACGAAGTTATGCTTTTTAGACATCTAAATCTA GG	Deletion of *clpX* gene in *S. gordonii*
ClpPpet20bF	GGAATTCCATATGATTCCTGTAGTTATTGAAC	Purification of ClpP
ClpPsmupMALc2B	CGCGGATCCTTATTTTAATTCATTATTTTCCATG	Purification of ClpP
ssrAVAAR	TAGGAATTCTTAGGCAGCTACTGCATAATTTTCAGAG<br>TAAGAATTGGTATTCTTGGCTGCTTTGTATAGTTCATCCATGC	Cloning of GFP+ssr-AVAA in pIB190
SsbA-Del6-190B	ACTGGATCCTTATGAAATATCCATAGGATTTG	Deletion of DivIVA C-terminal 6 residues
