# Supplementary material for: Strain-Dependent Recognition of a Unique Degradation Motif by ClpXP in Streptococcus mutans
Source: mSphere. 2016 Dec 7;1(6):e00287-16. doi: 10.1128/mSphere.00287-16 (PMC5143411; doi:10.1128/mSphere.00287-16)
Supplement: Table S2 [file sph006162201st2.pdf]

Table S2: Differential expression of protein spots in UA159 vs isogenic *ΔclpX* strain

Spot No.	pI	MW	Average WT Spot %	Average *ΔclpX* Spot %	WT *ΔclpX* Difference	WT vs *Δclpx* test(p)
*21	6.5	1,45,752	0.037	0.012	3.0	0.028
40	6.6	1,23,831	0.399	0.231	1.7	0.023
91	5.5	76,080	0.019	0.006	3.1	0.022
141	5.5	57,797	0.079	0.045	1.7	0.026
142	5.0	58,979	0.016	0.009	1.8	0.003
188	5.1	50,992	0.019	0.009	2.1	0.020
*194	5.3	51,476	0.202	0.010	21.2	0.000
229	6.5	46,960	1.162	0.659	1.8	0.044
312	6.2	41,015	0.287	0.154	1.9	0.017
*328	6.1	40,155	0.066	0.023	2.9	0.018
357	5.8	33,811	0.063	0.030	2.1	0.041
367	5.7	32,584	0.227	0.123	1.8	0.040
408	6.2	30,696	0.427	0.235	1.8	0.030
416	6.0	31,173	0.068	0.034	2.0	0.034
419	6.5	30,985	0.056	0.030	1.9	0.032
446	6.4	29,668	0.042	0.018	2.4	0.022
453	5.8	27,044	0.072	0.022	3.3	0.056
462	6.6	28,802	0.197	0.088	2.2	0.016
465	6.4	28,614	0.063	0.033	1.9	0.009
*501	5.1	22,071	0.061	0.029	2.1	0.005
522	6.3	20,295	0.190	0.048	4.0	0.024
531	6.7	19,316	0.102	0.044	2.3	0.021
532	6.5	18,864	0.322	0.081	4.0	0.001
544	5.4	17,093	0.181	0.093	1.9	0.018
545	6.0	17,062	0.061	0.020	3.0	0.029
*551	6.4	15,243	0.300	0.069	4.3	0.009
557	6.2	13,973	0.201	0.067	3.0	0.066
1	6.3	2,03,575	0.039	0.125	-3.2	0.018
*7	5.9	1,64,880	0.006	0.024	-3.7	0.007
*8	5.8	1,63,924	0.018	0.050	-2.7	0.014
34	6.2	1,28,089	0.017	0.098	-5.7	0.001
*36	6.3	1,26,812	0.031	0.242	-7.9	0.036
57	6.0	1,02,441	0.012	0.055	-4.7	0.004
82	6.1	81,671	0.008	0.025	-3.1	0.021
167	5.0	53,751	0.184	0.538	-2.9	0.006
*243	5.9	45,539	0.051	0.102	-2.0	0.037
251	6.6	45,109	0.025	0.066	-2.6	0.045
253	5.0	40,937	0.009	0.030	-3.4	0.017
*257	5.0	40,829	0.057	0.183	-3.2	0.003
*272	5.1	39,472	0.023	0.046	-2.0	0.035
307	5.7	38,947	0.057	0.094	-1.7	0.035
310	5.8	38,257	0.015	0.047	-3.2	0.034
*335	5.5	36,111	0.020	0.111	-5.5	0.008
*336	5.6	36,111	0.036	0.154	-4.2	0.014
384	6.1	36,209	0.013	0.024	-1.8	0.015
412	7.2	30,972	2.565	6.513	-2.5	0.019
*447	6.6	29,630	0.044	0.095	-2.2	0.024
*466	5.9	26,491	0.018	0.061	-3.4	0.017
482	4.9	24,911	0.195	0.354	-1.8	0.042
493	4.9	22,903	0.034	0.085	-2.5	0.038
*497	5.6	23,051	0.018	0.066	-3.6	0.124
516	5.2	18,851	0.059	0.118	-2.0	0.045
*546	6.3	17,020	0.417	0.849	-2.0	0.030

* denotes spots were selected for mass-spec identification (Table S3)
