# Supplementary material for: Strain-Dependent Recognition of a Unique Degradation Motif by ClpXP in Streptococcus mutans
Source: mSphere. 2016 Dec 7;1(6):e00287-16. doi: 10.1128/mSphere.00287-16 (PMC5143411; doi:10.1128/mSphere.00287-16)
Supplement: Table S3 [file sph006162201st3.pdf]

Table S3: Differential expression of proteins identifed by mass-spec

Spot no	Gene no.	Gene name	MW (kD)	pI	WT vs Δ*clpX* Difference
The proteins increased in Δ*clpX* compared to WT:					
7	SMU_1989	*rpoC*	136	6.37	3.7
8	SMU_08	*trcF*	134	5.68	2.7
36	SMU_689	*altA*	107	5.52	7.9
243	SMU_418	*nusA*	45	4.79	2.0
257	SMU_557	*divIVA*	31	4.46	3.2
272	SMU_714	Ef-Tu	44	4.84	2.0
335	SMU_24	*aspC*	43	5.38	5.5
336	SMU_2085	*recA*	41	5.23	4.2
447	SMU_1632	*pfs*	25	5.71	2.2
466	SMU_329	unknown	25	5.38	3.4
497	SMU_1859	*ssbA*	18	4.98	3.6
546	SMU_1496	*lacA*	16	6.07	2.0
The proteins decreased in Δ*clpX* compared to WT:					
21	SMU_1851	*uvrA*	104	6.42	- 3.0
194	SMU_949	*clpX*	45	4.72	- 21.2
328	SMU_852	*cpsY*	35	5.53	- 2.9
501	SMU_1947	*nusG*	20	4.61	- 2.1
551	SMU_1845	*nusB*	17	6.17	- 4.3
